# Supplementary material for: Computational Reconstruction of Clonal Hierarchies From Bulk Sequencing Data of Acute Myeloid Leukemia Samples
Source: Front Physiol. 2021 Aug 23;12:596194. doi: 10.3389/fphys.2021.596194 (PMC8419336; doi:10.3389/fphys.2021.596194)
Supplement: Supplementary file 1 [file Data_Sheet_1.PDF]

Supplementary Information

Computational reconstruction of clonal hierarchies from bulk sequencing data of acute myeloid leukemia samples

T. Stiehl, A. Marciniak-Czochra

Contents

1 Supplemental Figures **2**

2 Matrices, Data-Vectors and Solutions **4**

1 Supplemental Figures

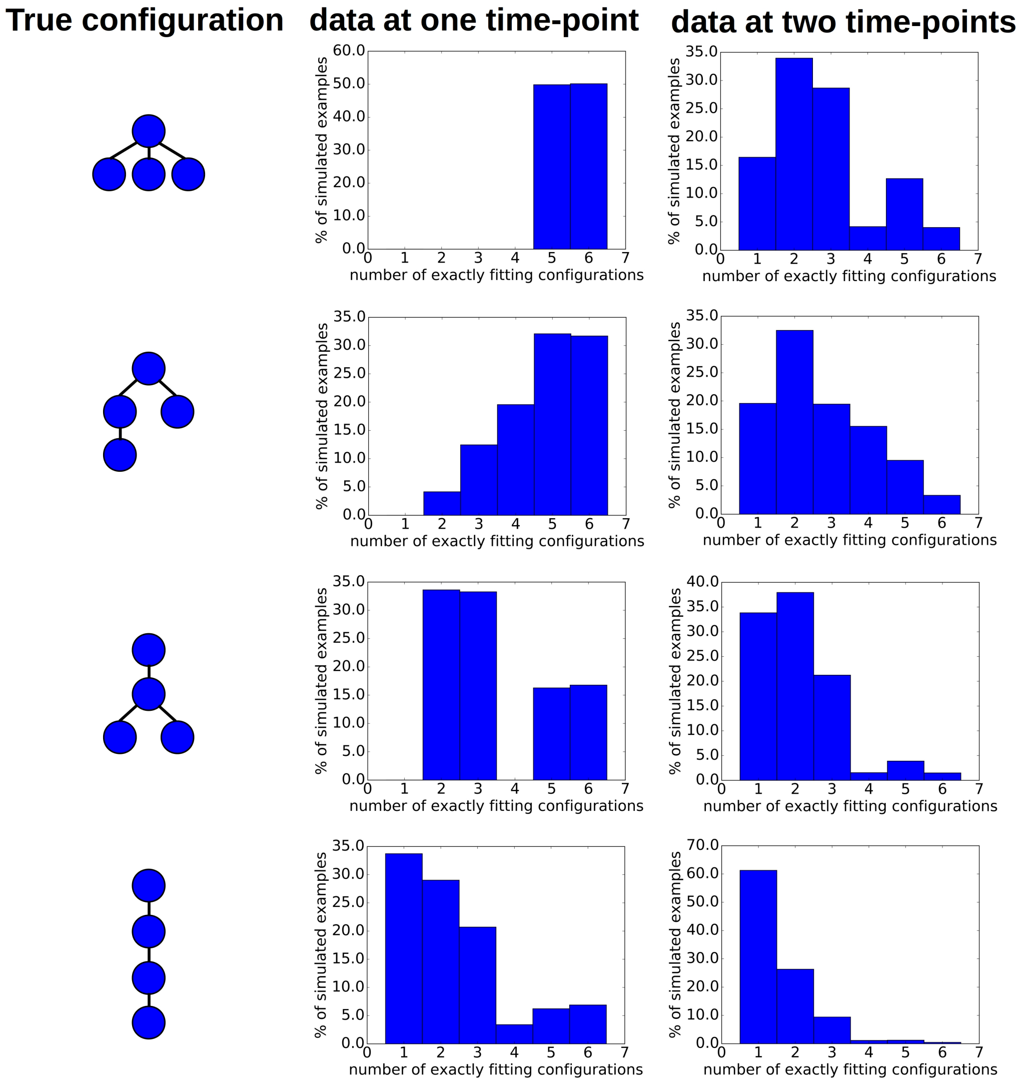

Supplemental Figure 1: Impact of the true hierarchy on the uniqueness of reconstructions. For each of the depicted "true configurations" shown in the first column bulk data were generated by assigning a random frequency to each of the clones. Our clonal reconstruction algorithm was applied to this numerically generated bulk data to assess how many different tree configurations are in line with it. This procedure was repeated 10000 times for each of the hierarchies. The second column quantifies in how many percent of the

10000 simulations only one, two, three etc. tree configurations could be fitted to the data without error. The data shown is based on 10000 reconstructions, each of which used one random bulk dataset as input. This corresponds to a clinical scenario where only data from diagnosis is available. The ambiguity of the reconstructed hierarchies can be reduced by taking into account data from multiple time-points, such as diagnosis and relapse. The third column quantifies the ambiguity of reconstructed hierarchies which are based on two randomly generated bulk datasets, corresponding to a clinical scenario where data from diagnosis and relapse are available. The simulations show that in case of a linear hierarchy the probability of a unique reconstitution is highest and in the case of a hierarchy of depth one it is lowest. ■

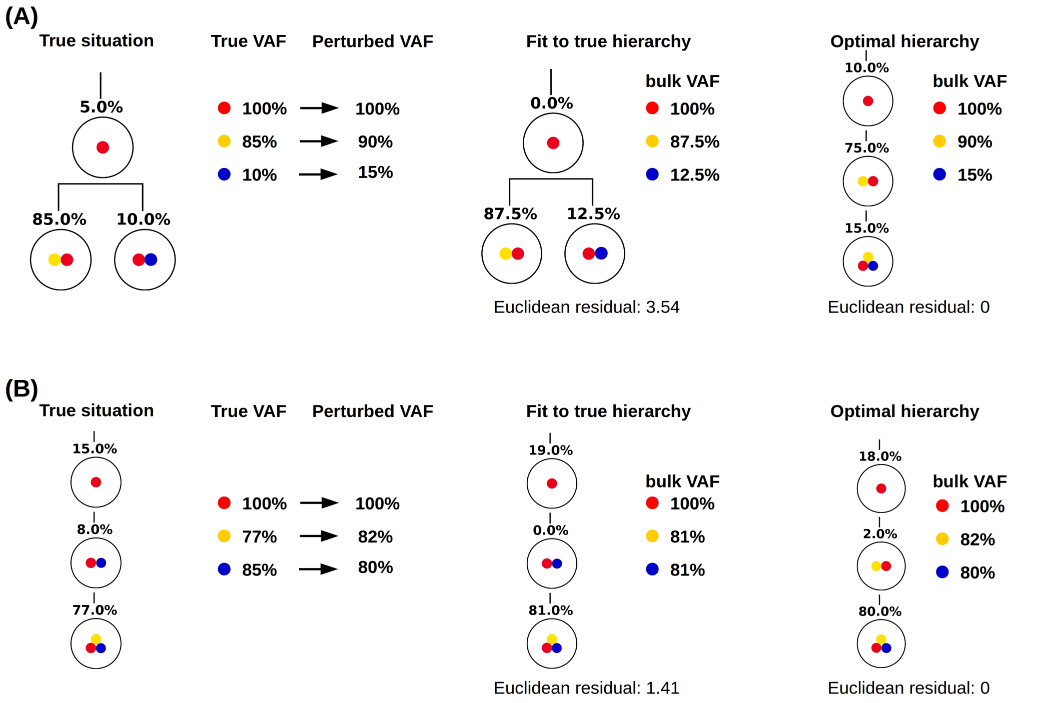

Supplemental Figure 2: In case of significant measurement errors the true configuration may not fit the data optimally. The figure shows two examples. On the left of each panel the true configuration is shown together with the resulting bulk variant allele frequencies (VAF). The VAFs are perturbed by ±5%. If the true hierarchy is fitted to the perturbed bulk VAFs, the data

cannot be exactly reproduced, as shown by the euclidean residuals. However, other tree configurations which are different from the true configuration can fit the data without error. These configurations are depicted on the right of each panel. ■

2 Matrices, Data-Vectors and Solutions

This Supplement lists the matrices A, right handsides b and solutions x of the system $Ax = b$ of all clonal hierarchies depicted in Figures 2-4 of the main text. The condition numbers with respect to the l^2 (κ_2), the infinity (κ_∞) and the Frobenius (κ_{fro}) norms are also provided.

Configurations Matching Data at Diagnosis (Fig. 2)

Patient 1

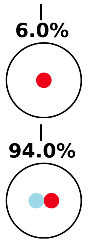

$$\begin{bmatrix} 1 & 1 \\ 0 & 1 \end{bmatrix} \cdot \begin{bmatrix} 6.0 \\ 94.0 \end{bmatrix} = \begin{bmatrix} 100 \\ 94 \end{bmatrix}$$

$\kappa_2(A) = 2.618$
$\kappa_{fro}(A) = 3.0$
$\kappa_\infty(A) = 4.0$

Patient 2

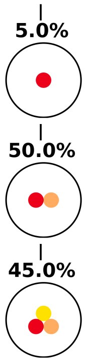

$$\begin{bmatrix} 1 & 1 & 1 \\ 0 & 1 & 1 \\ 0 & 0 & 1 \end{bmatrix} \cdot \begin{bmatrix} 5.0 \\ 50.0 \\ 45.0 \end{bmatrix} = \begin{bmatrix} 100 \\ 95 \\ 45 \end{bmatrix}$$

$\kappa_2(A) = 4.049$
$\kappa_{fro}(A) = 5.477$
$\kappa_\infty(A) = 6.0$

Patient 3

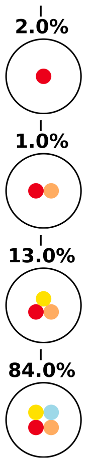

$$\begin{bmatrix}1 & 1 & 1 & 1\\0 & 1 & 1 & 1\\0 & 0 & 1 & 1\\0 & 0 & 0 & 1\end{bmatrix} \cdot \begin{bmatrix}2.0\\1.0\\13.0\\84.0\end{bmatrix} = \begin{bmatrix}100\\98\\97\\84\end{bmatrix}$$

$\kappa_2(A) = 5.411$
$\kappa_{fro}(A) = 8.367$
$\kappa_\infty(A) = 8.0$

Patient 4

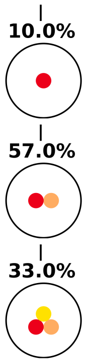

$$\begin{bmatrix}1 & 1 & 1\\0 & 1 & 1\\0 & 0 & 1\end{bmatrix} \cdot \begin{bmatrix}10.0\\57.0\\33.0\end{bmatrix} = \begin{bmatrix}100\\90\\33\end{bmatrix}$$

$\kappa_2(A) = 4.049$
$\kappa_{fro}(A) = 5.477$
$\kappa_\infty(A) = 6.0$

Patient 5

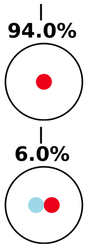

$$\begin{bmatrix} 1 & 1 \\ 0 & 1 \end{bmatrix} \cdot \begin{bmatrix} 94.0 \\ 6.0 \end{bmatrix} = \begin{bmatrix} 100 \\ 6 \end{bmatrix}$$

$\kappa_2(A) = 2.618$
$\kappa_{fro}(A) = 3.0$
$\kappa_\infty(A) = 4.0$

Patient 6

Possibility 1

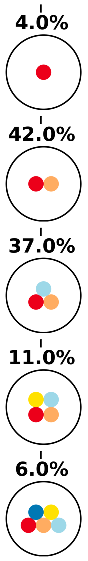

$$\begin{bmatrix}1 & 1 & 1 & 1 & 1\\0 & 1 & 1 & 1 & 1\\0 & 0 & 1 & 1 & 1\\0 & 0 & 0 & 1 & 1\\0 & 0 & 0 & 0 & 1\end{bmatrix} \cdot \begin{bmatrix}4.0\\42.0\\37.0\\11.0\\6.0\end{bmatrix} = \begin{bmatrix}100\\96\\54\\17\\6\end{bmatrix}$$

$\kappa_2(A) = 6.742$
$\kappa_{fro}(A) = 11.619$
$\kappa_\infty(A) = 10.0$

Possibility 2

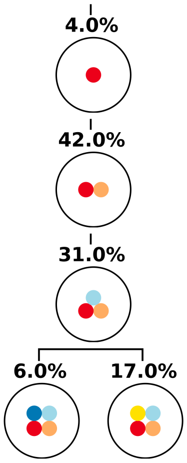

$$\begin{bmatrix}1 & 1 & 1 & 1 & 1\\0 & 1 & 1 & 1 & 1\\0 & 0 & 1 & 1 & 1\\0 & 0 & 0 & 1 & 0\\0 & 0 & 0 & 0 & 1\end{bmatrix} \cdot \begin{bmatrix}4.0\\42.0\\31.0\\6.0\\17.0\end{bmatrix} = \begin{bmatrix}100\\96\\54\\6\\17\end{bmatrix}$$

$\kappa_2(A) = 6.916$
$\kappa_{fro}(A) = 11.225$
$\kappa_\infty(A) = 15.0$

Possibility 3

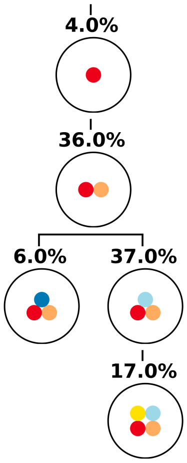

$$\begin{bmatrix} 1 & 1 & 1 & 1 & 1 \\ 0 & 1 & 1 & 1 & 1 \\ 0 & 0 & 1 & 0 & 0 \\ 0 & 0 & 0 & 1 & 1 \\ 0 & 0 & 0 & 0 & 1 \end{bmatrix} \cdot \begin{bmatrix} 4.0 \\ 36.0 \\ 6.0 \\ 37.0 \\ 17.0 \end{bmatrix} = \begin{bmatrix} 100 \\ 96 \\ 6 \\ 54 \\ 17 \end{bmatrix}$$

$\kappa_2(A) = 6.555$
$\kappa_{fro}(A) = 10.817$
$\kappa_\infty(A) = 15.0$

Possibility 4

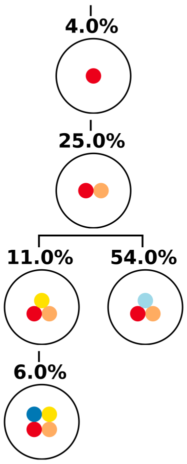

$$\begin{bmatrix} 1 & 1 & 1 & 1 & 1 \\ 0 & 1 & 1 & 1 & 1 \\ 0 & 0 & 1 & 1 & 0 \\ 0 & 0 & 0 & 1 & 0 \\ 0 & 0 & 0 & 0 & 1 \end{bmatrix} \cdot \begin{bmatrix} 4.0 \\ 25.0 \\ 11.0 \\ 6.0 \\ 54.0 \end{bmatrix} = \begin{bmatrix} 100 \\ 96 \\ 17 \\ 6 \\ 54 \end{bmatrix}$$

$\kappa_2(A) = 6.555$
$\kappa_{fro}(A) = 10.817$
$\kappa_\infty(A) = 15.0$

Possibility 5

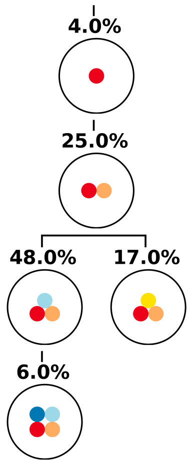

$$\begin{bmatrix}1 & 1 & 1 & 1 & 1\\0 & 1 & 1 & 1 & 1\\0 & 0 & 1 & 1 & 0\\0 & 0 & 0 & 1 & 0\\0 & 0 & 0 & 0 & 1\end{bmatrix} \cdot \begin{bmatrix}4.0\\25.0\\48.0\\6.0\\17.0\end{bmatrix} = \begin{bmatrix}100\\96\\54\\6\\17\end{bmatrix}$$

$\kappa_2(A) = 6.555$
$\kappa_{fro}(A) = 10.817$
$\kappa_\infty(A) = 15.0$

Possibility 6

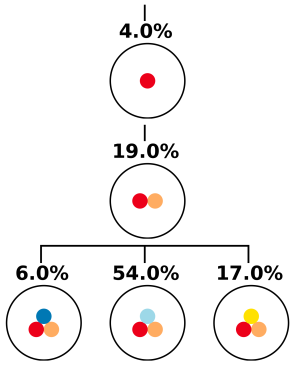

$$\begin{bmatrix}1 & 1 & 1 & 1 & 1\\0 & 1 & 1 & 1 & 1\\0 & 0 & 1 & 0 & 0\\0 & 0 & 0 & 1 & 0\\0 & 0 & 0 & 0 & 1\end{bmatrix} \cdot \begin{bmatrix}4.0\\19.0\\6.0\\54.0\\17.0\end{bmatrix} = \begin{bmatrix}100\\96\\6\\54\\17\end{bmatrix}$$

$\kappa_2(A) = 6.846$
$\kappa_{fro}(A) = 10.392$
$\kappa_\infty(A) = 20.0$

Configurations Matching Data at Relapse (Fig. 3)

Patient 1

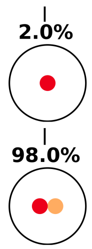

$$\begin{bmatrix}1 & 1\\0 & 1\end{bmatrix} \cdot \begin{bmatrix}2.0\\98.0\end{bmatrix} = \begin{bmatrix}100\\98\end{bmatrix}$$

$\kappa_2(A) = 2.618$
$\kappa_{fro}(A) = 3.0$
$\kappa_\infty(A) = 4.0$

Patient 2

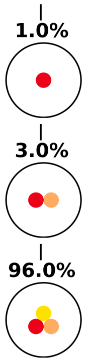

$$\begin{bmatrix} 1 & 1 & 1 \\ 0 & 1 & 1 \\ 0 & 0 & 1 \end{bmatrix} \cdot \begin{bmatrix} 1.0 \\ 3.0 \\ 96.0 \end{bmatrix} = \begin{bmatrix} 100 \\ 99 \\ 96 \end{bmatrix}$$

$\kappa_2(A) = 4.049$
$\kappa_{fro}(A) = 5.477$
$\kappa_\infty(A) = 6.0$

Patient 3

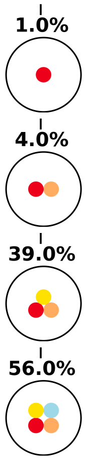

$$\begin{bmatrix}1 & 1 & 1 & 1\\0 & 1 & 1 & 1\\0 & 0 & 1 & 1\\0 & 0 & 0 & 1\end{bmatrix} \cdot \begin{bmatrix}1.0\\4.0\\39.0\\56.0\end{bmatrix} = \begin{bmatrix}100\\99\\95\\56\end{bmatrix}$$

$\kappa_2(A) = 5.411$
$\kappa_{fro}(A) = 8.367$
$\kappa_\infty(A) = 8.0$

Patient 4

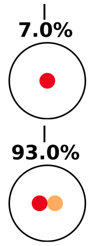

$$\begin{bmatrix}1 & 1\\0 & 1\end{bmatrix} \cdot \begin{bmatrix}7.0\\93.0\end{bmatrix} = \begin{bmatrix}100\\93\end{bmatrix}$$

$\kappa_2(A) = 2.618$
$\kappa_{fro}(A) = 3.0$
$\kappa_\infty(A) = 4.0$

Patient 5

Possibility 1

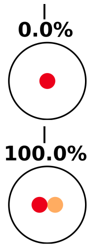

$$\begin{bmatrix} 1 & 1 \\ 0 & 1 \end{bmatrix} \cdot \begin{bmatrix} 0.0 \\ 100.0 \end{bmatrix} = \begin{bmatrix} 100 \\ 100 \end{bmatrix}$$

$\kappa_2(A) = 2.618$
$\kappa_{fro}(A) = 3.0$
$\kappa_\infty(A) = 4.0$

Possibility 2

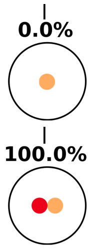

$$\begin{bmatrix} 1 & 1 \\ 0 & 1 \end{bmatrix} \cdot \begin{bmatrix} 0.0 \\ 100.0 \end{bmatrix} = \begin{bmatrix} 100 \\ 100 \end{bmatrix}$$

$\kappa_2(A) = 2.618$
$\kappa_{fro}(A) = 3.0$
$\kappa_\infty(A) = 4.0$

Patient 6

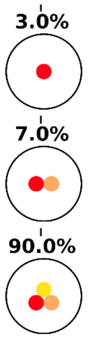

$$\begin{bmatrix}1 & 1 & 1\\0 & 1 & 1\\0 & 0 & 1\end{bmatrix} \cdot \begin{bmatrix}3.0\\7.0\\90.0\end{bmatrix} = \begin{bmatrix}100\\97\\90\end{bmatrix}$$

$\kappa_2(A) = 4.049$
$\kappa_{fro}(A) = 6.0$
$\kappa_\infty(A) = 5.477$

Configurations Matching Data at Diagnosis and Relapse (Fig. 4)

Patient 1

Diagnosis:

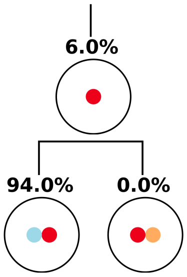

$$\begin{bmatrix}1 & 1 & 1\\0 & 1 & 0\\0 & 0 & 1\end{bmatrix} \cdot \begin{bmatrix}6.0\\94.0\\0.0\end{bmatrix} = \begin{bmatrix}100\\94\\0\end{bmatrix}$$

Relapse:

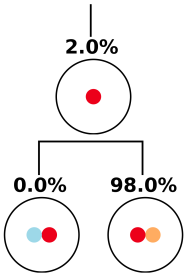

$$\begin{bmatrix}1 & 1 & 1\\0 & 1 & 0\\0 & 0 & 1\end{bmatrix} \cdot \begin{bmatrix}2.0\\0.0\\98.0\end{bmatrix} = \begin{bmatrix}100\\0\\98\end{bmatrix}$$

$\kappa_2(A) = 3.732$
$\kappa_{fro}(A) = 5.0$
$\kappa_\infty(A) = 9.0$

Patient 2

Diagnosis:

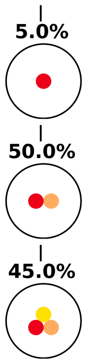

$$\begin{bmatrix}1 & 1 & 1\\0 & 1 & 1\\0 & 0 & 1\end{bmatrix} \cdot \begin{bmatrix}5.0\\50.0\\45.0\end{bmatrix} = \begin{bmatrix}100\\95\\45\end{bmatrix}$$

Relapse:

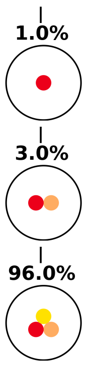

$$\begin{bmatrix}1 & 1 & 1\\0 & 1 & 1\\0 & 0 & 1\end{bmatrix} \cdot \begin{bmatrix}1.0\\3.0\\96.0\end{bmatrix} = \begin{bmatrix}100\\99\\96\end{bmatrix}$$

$\kappa_2(A) = 4.049$
$\kappa_{fro}(A) = 5.477$
$\kappa_\infty(A) = 6.0$

Patient 3

Diagnosis:

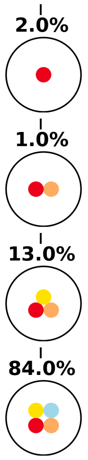

$$\begin{bmatrix} 1 & 1 & 1 & 1 \\ 0 & 1 & 1 & 1 \\ 0 & 0 & 1 & 1 \\ 0 & 0 & 0 & 1 \end{bmatrix} \cdot \begin{bmatrix} 2.0 \\ 1.0 \\ 13.0 \\ 84.0 \end{bmatrix} = \begin{bmatrix} 100 \\ 98 \\ 97 \\ 84 \end{bmatrix}$$

Relapse:

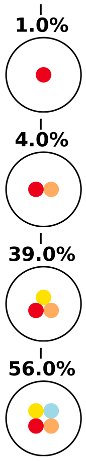

$$\begin{bmatrix} 1 & 1 & 1 & 1 \\ 0 & 1 & 1 & 1 \\ 0 & 0 & 1 & 1 \\ 0 & 0 & 0 & 1 \end{bmatrix} \cdot \begin{bmatrix} 1.0 \\ 4.0 \\ 39.0 \\ 56.0 \end{bmatrix} = \begin{bmatrix} 100 \\ 99 \\ 95 \\ 56 \end{bmatrix}$$

$\kappa_2(A) = 5.411$
$\kappa_{fro}(A) = 8.367$
$\kappa_\infty(A) = 8.0$

Patient 4

Diagnosis:

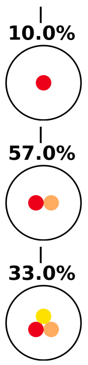

$$\begin{bmatrix} 1 & 1 & 1 \\ 0 & 1 & 1 \\ 0 & 0 & 1 \end{bmatrix} \cdot \begin{bmatrix} 10.0 \\ 57.0 \\ 33.0 \end{bmatrix} = \begin{bmatrix} 100 \\ 90 \\ 33 \end{bmatrix}$$

Relapse:

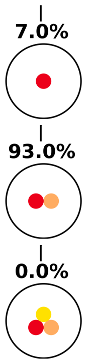

$$\begin{bmatrix} 1 & 1 & 1 \\ 0 & 1 & 1 \\ 0 & 0 & 1 \end{bmatrix} \cdot \begin{bmatrix} 7.0 \\ 93.0 \\ 0.0 \end{bmatrix} = \begin{bmatrix} 100 \\ 93 \\ 0 \end{bmatrix}$$

$\kappa_2(A) = 4.049$
$\kappa_{fro}(A) = 5.477$
$\kappa_\infty(A) = 6.0$

Patient 5

Diagnosis:

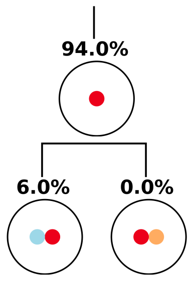

$$\begin{bmatrix}1 & 1 & 1\\0 & 1 & 0\\0 & 0 & 1\end{bmatrix} \cdot \begin{bmatrix}94.0\\6.0\\0.0\end{bmatrix} = \begin{bmatrix}100\\6\\0\end{bmatrix}$$

Relapse:

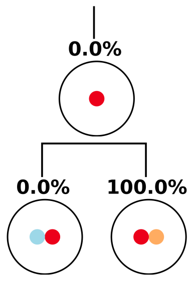

$$\begin{bmatrix}1 & 1 & 1\\0 & 1 & 0\\0 & 0 & 1\end{bmatrix} \cdot \begin{bmatrix}0.0\\0.0\\100.0\end{bmatrix} = \begin{bmatrix}100\\0\\100\end{bmatrix}$$

$\kappa_2(A) = 3.732$
$\kappa_{fro}(A) = 5.0$
$\kappa_\infty(A) = 9.0$

Patient 6

Possibility 1

Diagnosis:

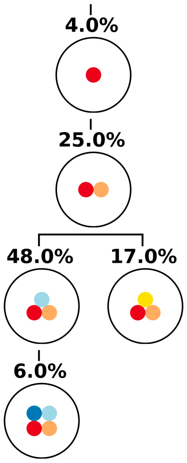

$$\begin{bmatrix} 1 & 1 & 1 & 1 & 1 \\ 0 & 1 & 1 & 1 & 1 \\ 0 & 0 & 1 & 1 & 0 \\ 0 & 0 & 0 & 1 & 0 \\ 0 & 0 & 0 & 0 & 1 \end{bmatrix} \cdot \begin{bmatrix} 4.0 \\ 25.0 \\ 48.0 \\ 6.0 \\ 17.0 \end{bmatrix} = \begin{bmatrix} 100 \\ 96 \\ 54 \\ 6 \\ 17 \end{bmatrix}$$

Relapse:

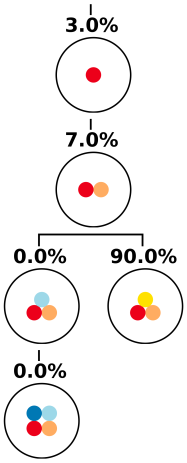

$$\begin{bmatrix} 1 & 1 & 1 & 1 & 1 \\ 0 & 1 & 1 & 1 & 1 \\ 0 & 0 & 1 & 1 & 0 \\ 0 & 0 & 0 & 1 & 0 \\ 0 & 0 & 0 & 0 & 1 \end{bmatrix} \cdot \begin{bmatrix} 3.0 \\ 7.0 \\ 0.0 \\ 0.0 \\ 90.0 \end{bmatrix} = \begin{bmatrix} 100 \\ 97 \\ 0 \\ 0 \\ 90 \end{bmatrix}$$

$\kappa_2(A) = 6.555$
$\kappa_{fro}(A) = 10.817$
$\kappa_\infty(A) = 15.0$

Possibility 2

Diagnosis:

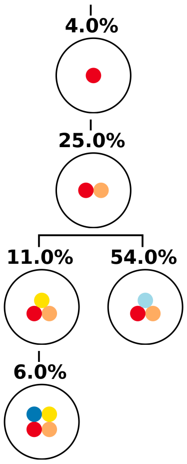

$$\begin{bmatrix} 1 & 1 & 1 & 1 & 1 \\ 0 & 1 & 1 & 1 & 1 \\ 0 & 0 & 1 & 1 & 0 \\ 0 & 0 & 0 & 1 & 0 \\ 0 & 0 & 0 & 0 & 1 \end{bmatrix} \cdot \begin{bmatrix} 4.0 \\ 25.0 \\ 11.0 \\ 6.0 \\ 54.0 \end{bmatrix} = \begin{bmatrix} 100 \\ 96 \\ 17 \\ 6 \\ 54 \end{bmatrix}$$

Relapse:

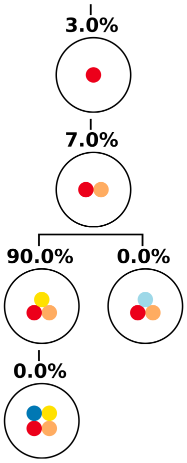

$$\begin{bmatrix}1 & 1 & 1 & 1 & 1\\0 & 1 & 1 & 1 & 1\\0 & 0 & 1 & 1 & 0\\0 & 0 & 0 & 1 & 0\\0 & 0 & 0 & 0 & 1\end{bmatrix} \cdot \begin{bmatrix}3.0\\7.0\\90.0\\0.0\\0.0\end{bmatrix} = \begin{bmatrix}100\\97\\90\\0\\0\end{bmatrix}$$

$\kappa_2(A) = 6.555$
$\kappa_{fro}(A) = 10.817$
$\kappa_\infty(A) = 15.0$

Possibility 3

Diagnosis:

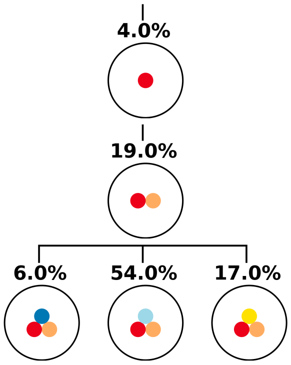

$$\begin{bmatrix}1 & 1 & 1 & 1 & 1\\0 & 1 & 1 & 1 & 1\\0 & 0 & 1 & 0 & 0\\0 & 0 & 0 & 1 & 0\\0 & 0 & 0 & 0 & 1\end{bmatrix} \cdot \begin{bmatrix}4.0\\19.0\\6.0\\54.0\\17.0\end{bmatrix} = \begin{bmatrix}100\\96\\6\\54\\17\end{bmatrix}$$

Relapse:

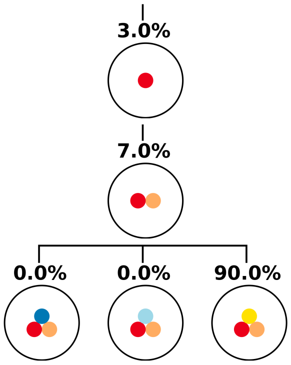

$$\begin{bmatrix} 1 & 1 & 1 & 1 & 1 \\ 0 & 1 & 1 & 1 & 1 \\ 0 & 0 & 1 & 0 & 0 \\ 0 & 0 & 0 & 1 & 0 \\ 0 & 0 & 0 & 0 & 1 \end{bmatrix} \cdot \begin{bmatrix} 3.0 \\ 7.0 \\ 0.0 \\ 0.0 \\ 90.0 \end{bmatrix} = \begin{bmatrix} 100 \\ 97 \\ 0 \\ 0 \\ 90 \end{bmatrix}$$

$\kappa_2(A) = 6.846$
$\kappa_{fro}(A) = 10.392$
$\kappa_\infty(A) = 20.0$
